# Enhancement of Chondrogenic Differentiation in Bone Marrow-Derived Stem Cell Spheroids by *Cuminum cyminum* Methanolic Extract: Insights into Concentration-Dependent mRNA Expression and Gene Clustering Analysis

**DOI:** 10.3390/jpm14121142

**Published:** 2024-12-05

**Authors:** Kyung-Hwan Na, Hyun-Jin Lee, Ju-Hwan Kim, Md. Salah Uddin, Yoon-Hee Park, Young-Min Song, Chul-Sung Park, Jun-Beom Park

**Affiliations:** 1 Department of Medicine, Graduate School, The Catholic University of Korea, Seoul 06591, Republic of Korea; nabali8@gmail.com; 2 Department of Periodontics, College of Medicine, The Catholic University of Korea, Seoul 06591, Republic of Korea; hyunjinlee0423@gmail.com (H.-J.L.); juhwank33@naver.com (J.-H.K.); quasar2813@snu.ac.kr (Y.-M.S.); 3 Ethnobotanical Database of Bangladesh, Tejgaon, Dhaka 1208, Bangladesh; plantsofbd@gmail.com; 4 Ebiogen, Seoul 04785, Republic of Korea; 5 Department of Biomedical Science, Graduate School of Biomedical Science & Engineering, Hanyang University, Seoul 04763, Republic of Korea; chulsung12@gmail.com; 6 Dental Implantology, Graduate School of Clinical Dental Science, The Catholic University of Korea, Seoul 06591, Republic of Korea

**Keywords:** cell differentiation, chondrogenesis, Cuminum, herbal medicine, medical plants, stem cells

## Abstract

**Background/Objectives**: *Cuminum cyminum* L. has been utilized as a medicinal plant for centuries. This research sought to examine the effects of cumin methanolic extract (CMT) on the chondrogenic differentiation of human bone marrow-derived mesenchymal stem cells. **Methods**: Spheroids were generated using human stem cells and cultured with CMT at concentrations between 0 and 1 µg/mL. Morphological assessments and cell viability tests were conducted on days 1 and 3. Chondrogenic differentiation expression was evaluated through quantitative polymerase chain reaction, Western blot, and RNA sequencing. SOX9, FAM20B, COL2A1, and COL1A1 mRNA expression levels were determined using real-time polymerase chain reaction. Protein expression was analyzed via Western blot. **Results**: Throughout this study, the spheroids maintained their integrity and shape. No significant variations in spheroid diameter were observed among the groups. CMT treatment enhanced the expression of SOX9 and FAM20B. **Conclusions**: The methanolic extract of *Cuminum cyminum* facilitated chondrogenic differentiation in human bone marrow-derived mesenchymal stem cells by modulating SOX9 and FAM20B expression. This indicates its potential application in cartilage tissue engineering.

## 1. Introduction

Stem cells have long been a subject of great interest, particularly in the treatment of various diseases [1,2,3]. Their unique properties include the ability to differentiate into a wide range of tissue types [4]. Additionally, stem cells secrete various paracrine factors, such as growth factors, which influence surrounding tissues [5,6]. In recent years, stem cells have been increasingly applied in tissue regeneration [7]. For example, mesenchymal stem cells (MSCs) have been directed towards chondrogenic differentiation in both in vitro and in vivo models, with promising clinical applications [8].

Three-dimensional (3D) cell culture techniques, such as spheroid formation, are widely used in regenerative medicine, particularly in cartilage repair [9]. The spheroid formation of multicellular MSCs has been reported to offer several benefits, including enhanced chondrogenesis and reduced fibrosis. This suggests that spheroid-based approaches may hold great promise for regenerating hyaline-like cartilage [10]. In a previous study, spheroids of MSCs derived from human gingival fibroblasts were generated on chitosan membranes, resulting in enhanced chondrogenic differentiation via the activation of the Rho/Rho-associated protein kinase pathway [11].

*Cuminum cyminum* L. (cumin) has been used as a medicinal plant since ancient times [12], recognized for its versatility in treating various ailments across different cultures [13]. Cumin supplements have been employed to regulate fasting blood sugar and serum insulin levels [14], and oral applications of cumin essential oil have demonstrated immunomodulatory effects [15]. Previous studies have shown that cumin extract enhances the osteogenic differentiation of bone marrow-derived stem cells by regulating the expression of RUNX2, BSP, and OCN [13,16]. Moreover, cumin components have also been applied in chondrogenesis and cartilage repair [17,18]. However, chondrocytes do not maintain chondrogenic differentiation in conventional two-dimensional cultures [19]. A previous study utilized MSC spheroids to evaluate the effects of growth factors on chondrogenic differentiation, with spheroids demonstrating superior cell survival and differentiation potential compared to single-cell suspensions [20]. This study aims to explore the effects of CMT on the chondrogenic differentiation of human mesenchymal stem cells using a spheroid culture model.

## 2. Materials and Methods

### 2.1. Preparation of Plant Materials

Cumin was collected from Shibgonj sub-district, Bogra district, Rajshahi division, Bangladesh by Md. Salah Uddin. A voucher specimen was labeled KRIB 0086021 at the Korea Research Institute of Bioscience and Biotechnology. The cumin seeds were dried and then ground. Subsequently, 75.0 g of the powder was extracted using 1 L of 99.9% (*v*/*v*) methanol, which was repeatedly sonicated for 15 min and then rested for 2 h, followed by 3 days of heating at 45 °C. Non-fluorescent cotton filters were utilized to strain the resulting product, and the filtered materials were concentrated using a speed vacuum concentrator (Hyper-VC2200, Hanil Science Co., Ltd., Seoul, Republic of Korea) under reduced pressure at 40 °C. This process yielded 13.2 g of CMT.

### 2.2. Design of This Present Study Utilizing Bone Marrow Mesenchymal Stem Cells

This present study protocol was evaluated and sanctioned by the Institutional Review Board of Seoul St Mary’s Hospital, College of Medicine, the Catholic University of Korea (KC20SISE0735; approval date: 17 September 2020). The research adhered to the principles and regulations outlined in the Declaration of Helsinki.

Bone marrow mesenchymal stem cells (BMSCs; Catholic MASTER cells) were supplied by the Catholic Institute of Cell Therapy (Seoul, Republic of Korea). Informed consent was obtained from the participants. The isolation and characterization of the BMSCs were conducted according to previously reported methods [21]. The cells were seeded on a culture dish and raised at 37 °C in an incubator with 95% air and 5% CO_2_, and the culture media was changed every one to two days.

### 2.3. Fabrication of Stem Cell Spheroids

To establish the control group, the investigators plated stem cells into concave microwells 600 µm in diameter (StemFIT 3D; MicroFIT, Seongnam-si, Gyeonggi-do, Republic of Korea) at a density of 1 × 10^6^ cells/well and cultured them in chondrogenic induction medium (STEMPRO chondrogenesis differentiation kit; Gibco, Grand Island, NY, USA) without the addition of CMT [22]. These concave microwell plates are designed to facilitate natural cell aggregation and uniform spheroid formation without the need for rotational incubation. This static culture environment supports consistent spheroid morphology and 3D differentiation studies by promoting natural cell–cell interactions.

Culture media were utilized to dissolve the product in the treatment groups. The researchers employed a range of concentrations, from 0.001 to 1 μg/mL, to investigate the effects of CMT, with the aim of identifying the optimal concentration. The final concentrations of CMT in the treatment groups were 0.001, 0.01, 0.1, and 1 μg/mL. A 0 µg/mL CMT group was prepared using culture media at the same concentration as the treatment groups, excluding the CMT concentration, and served as the control. On the first, third, seventh, and fourteenth days, the investigators conducted morphological evaluations using an inverted microscope (CKX41SF, Olympus Corporation, Tokyo, Japan). The diameters of the spheroids were measured on days 1, 3, 7, and 14 based on comparisons to a reference length [23,24].

### 2.4. Determination of Cellular Viability

The assessment of cellular viability was carried out using the CCK-8 assay kit (Dojindo, Tokyo, Japan) in accordance with the manufacturer’s instructions on days 1, 3, 7, and 14 [25]. The process involved incubating cell spheroids with tetrazolium monosodium salt for one hour at 37 °C, followed by the measurement of absorbance at 450 nm using a micro-plate reader (BioTek Instruments Inc., Winooski, VT, USA). Cellular viability was assessed in triplicate for each condition, adhering to standardized procedures to ensure consistency and minimize variability.

### 2.5. Evaluation of SOX9, FAM20B, COL2A1, and COL1A1 by Quantitative Real-Time Polymerase Chain Reaction (PCR)

Quantitative real-time polymerase chain reaction (qPCR) was conducted to assess the expression levels of SOX9, FAM20B, COL2A1, and COL1A1 in spheroids following 14 days of chondrogenic induction. Total RNA was extracted from the spheroids, and complementary DNA (cDNA) synthesis was performed using SuperScript II RTase (Invitrogen, Carlsbad, CA, USA) as per the manufacturer’s instructions. The mRNA levels of the chondrogenic markers (SOX9, FAM20B, COL2A1) and the fibrocartilage marker (COL1A1) were evaluated using the StepOnePlus™ Real-Time PCR System (Applied Biosystems, Foster City, CA, USA) and a SYBR Green PCR Kit (Applied Biosystems). The primer sequences used for qPCR were as follows: SOX9 (forward 5′-CCCTTCAACCTCCCACACT-3′, reverse 5′-GAGTTCTGGTGGTCGGTGTA-3′), FAM20B (forward 5′-CTAGTATGCTCATCCTTC-3′, reverse 5′-GTAGTTCAGTCTGTTCCAGGTG-3′), COL2A1 (forward 5′-AAGGTTTTCTGCAACATGGA-3′, reverse 5′-TCTTCTTGGGAACGTTTGCT-3′), and β-actin (forward 5′-AATGCTTCTAGGCGGACTATGA-3′, reverse 5′-TTTCTGCGCAAGTTAGGTTTT-3′). The qPCR protocol consisted of an initial denaturation at 95 °C for 10 min, followed by 40 cycles at 95 °C for 15 s and 59 °C for 30 s. The expression levels of the target genes were normalized to the reference gene β-actin, and the relative quantification was calculated using the 2^−ΔΔC_T_^ method, where ΔC_T_ represents the difference between the C_T_ values of the target and reference genes, and ΔΔC_T_ represents the difference between the ΔC_T_ of the sample and that of the control group. This approach allowed for the accurate determination of fold changes in gene expression, facilitating reliable comparisons across experimental groups. Gene expression analysis was performed in triplicate.

### 2.6. Western Blot

After 14 days of culturing spheroids in chondrogenic media, 300 spheroids were collected and washed in 1× phosphate-buffered saline. The cells were then lysed using RIPA buffer containing 50 mM Tris-HCl (pH 7.8), 150 mM NaCl, 1% IGEPAL, 10 mM NaF, 0.1 mM EDTA, and a protease inhibitor cocktail. Protein concentration was determined using the Bradford assay solution (Bio-Rad, Contra Costa County, CA, USA). Equal amounts of protein (10 µg) were separated with 10% sodium dodecyl sulfate-polyacrylamide gel electrophoresis (SDS-PAGE) for 2 h at 110 V and transferred onto PVDF membranes (Merck Millipore, Burlington, MA, USA) for 1.5 h at 100 V.

The membranes were blocked with 5% skimmed milk for 1 h at room temperature and then incubated overnight at 4 °C with primary antibodies at 1:1000 dilution, including anti-rabbit SOX9 antibody (D8G8H; Cell Signaling Technology, Inc., Danvers, MA, USA), anti-rabbit collagen II antibody (ab34712; Abcam, Cambridge, MA, USA), anti-rabbit collagen I antibody (ab34710; Abcam), and anti-mouse β-actin (SC-47778; Santa Cruz Biotechnology, Dallas, TX, USA).

Following primary antibody incubation, the membranes were washed and incubated with the appropriate secondary antibodies for 1 h at room temperature at a 1:3,000 dilution. The secondary antibodies used included m-IgGκ BP-HRP (Anti-Mouse) (Santa Cruz, SC-516102) and mouse anti-rabbit IgG-HRP (Santa Cruz, SC-2357). Visualization was achieved using the ECL Prime Western blotting reagent (RPN2232; GE Healthcare, Chicago, IL, USA), and images were acquired using X-ray film and a chemiluminescence Western blot system (Advansta, San Jose, CA, USA).

The relative protein expression levels were normalized to the corresponding loading control, β-actin. Each experiment was performed in triplicate, and quantifications were conducted using image analysis software (ImageJ, 1.53e, Bethesda, MD, USA) [26].

### 2.7. RNA Extraction, Sequencing Process and Analysis

Total RNA was extracted using the Trizol reagent (Invitrogen). The quality of the RNA was evaluated by the TapeStation 4000 System (Agilent Technologies, Amstelveen, the Netherlands), and RNA quantification was performed using the ND-2000 Spectrophotometer (Thermo Inc., Wilmington, DE, USA). The NEBNext Ultra II Directional RNA-Seq Kit (NEW ENGLAND BioLabs, Inc., Hitchin, UK) was used to prepare libraries from the total RNA, and the Poly(A) RNA Selection Kit (LEXOGEN, Inc., Vienna, Austria) was used to isolate mRNA. The isolated mRNA was used for cDNA synthesis and shearing, following the manufacturer’s instructions. Illumina indexes 1–12 were used for indexing, and the enrichment step was carried out. The mean fragment size was evaluated using TapeStation HS D1000 Screen Tape (Agilent Technologies, Amstelveen, The Netherlands), and the library quantification kit was used to perform quantification using a StepOne Real-Time PCR System (Life Technologies, Inc., Carlsbad, CA, USA). The NovaSeq 6000 (Illumina, Inc., San Diego, CA, USA) was used to perform high-throughput sequencing as paired-end 100 sequencing. The raw sequencing data were subjected to quality control using FastQC [27], and adapter and low-quality reads (<Q20) were removed using FASTX_Trimmer [28,29] and BBMap [30]. The trimmed reads were mapped to the reference genome using TopHat [28]. The read count data were processed based on fragments per kb per million (FPKM) reads and the geometric normalization method using EdgeR within R [31]. FPKM values were estimated using Cufflinks [32]. The genes with differential expression were subjected to pathway analysis using the Kyoto Encyclopedia of Genes and Genomes (KEGG) mapping software (version 5.0, Kanehisa Laboratories, Kyoto, Japan) [33]. Data mining and graphic visualization were performed using ExDEGA (Ebiogen Inc., Seoul, Republic of Korea).

### 2.8. Statistical Analysis

Statistical analyses were conducted in accordance with a previously published report [34]. The data are presented as the mean ± standard deviation of the experiments. Normality tests were conducted, and a one-way analysis of variance with post hoc Tukey’s test was employed to evaluate the differences between the groups using a commercial statistics program (SPSS 12 for Windows, SPSS Inc., Chicago, IL, USA). A *p*-value less than 0.05 was considered statistically significant.

## 3. Results

### 3.1. Assessment of Cell Morphology

**Figure 1:**
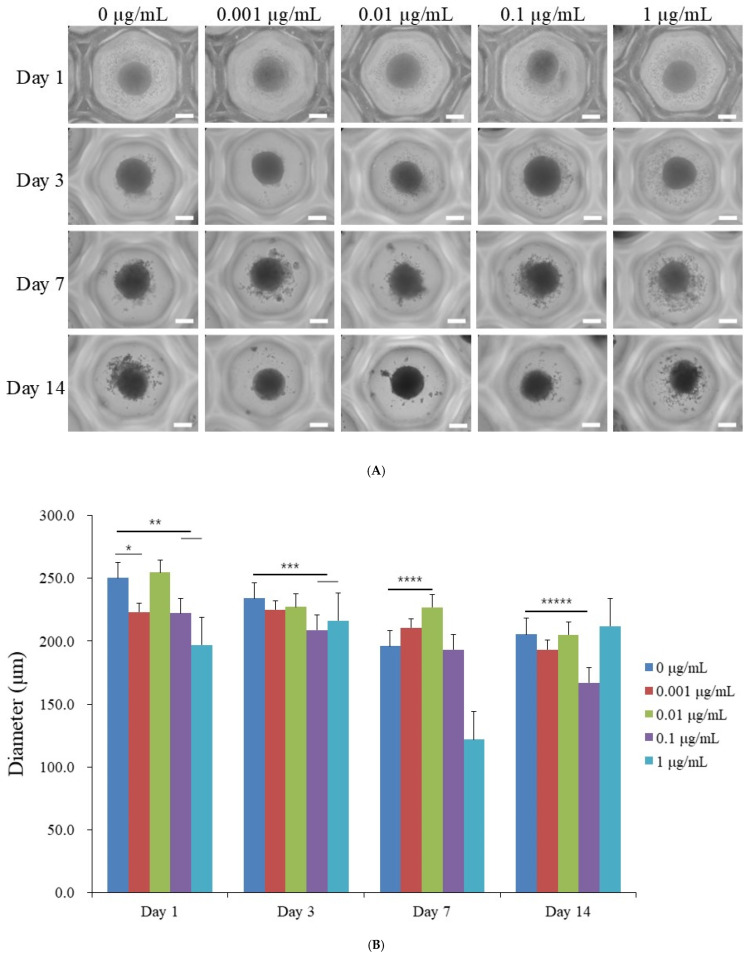

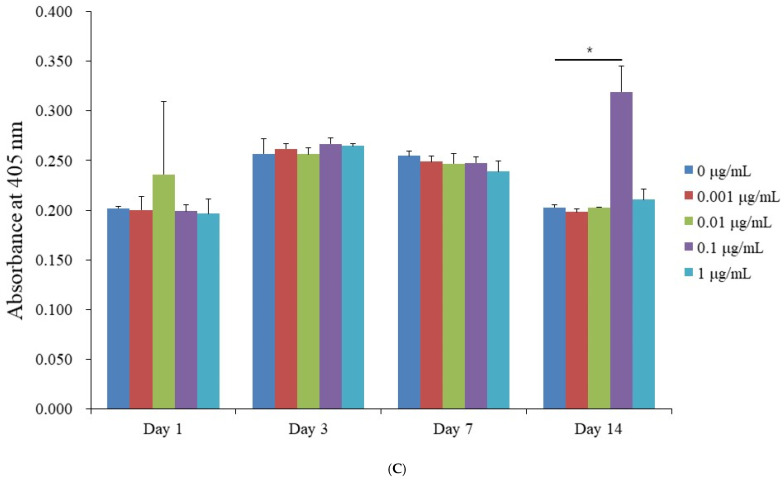
Evaluation of morphology and cellular viability of stem cell spheroids. (**A**) Assessment of cell morphology on days 1, 3, 7, and 14 for various concentrations of cumin methanolic extract, as seen under 100× original magnification. The scale bar in the image represents 200 μm. (**B**) Diameters of the cell spheroids on days 1, 3, 7, and 14 for different concentrations of the cumin methanolic extract. *: There was statistical difference when compared with the control on day 1. **: Statistical differences were noted when compared with the control on day 1. ***: There was statistical difference when compared with the control on day 3. ****: Statistical differences were noted when compared with the control on day 7. *****: There was statistical difference when compared with the control on day 14. (**C**) Cellular viability of the spheroids using the Cell Counting Kit-8 on days 1, 3, 7, and 14 for various concentrations of the extract. *: There was a significantly higher value for the 1 μg/mL group when compared with the control group on day 14.

The morphology of stem cells treated with CMT at final concentrations of 0, 0.001, 0.01, 0.1, and 1 μg/mL on days 1, 3, 7, and 14 is depicted in Figure 1A. On day 1, the spheroids were well formed in the microwells, and there were no noticeable differences compared to the untreated control. Extended incubation until day 14 did not reveal any morphological changes.

### 3.2. Measurement of Spheroid Diameters

Spheroids in all groups retained their overall morphology and exhibited minimal size changes over time. However, a minor but statistically significant reduction in diameter was observed in the 1 μg/mL CMT group on days 7 and 14 (*p* < 0.05), suggesting a concentration-dependent effect (Figure 1B). On day 1, the average spheroid diameters ranged from 197.1 μm to 254.7 μm across all groups. Despite the observed size reduction at higher CMT concentrations, spheroid morphology and structural integrity remained stable throughout the 14-day culture period (Figure 1).

### 3.3. Evaluation of Cellular Viability

The quantitative values for cellular viability on days 1, 3, 7, and 14 are shown in Figure 1C. The absorbance values at 450 nm for CMT concentrations of 0, 0.001, 0.01, 0.1, and 1 μg/mL on day 1 were 0.202 ± 0.002, 0.200 ± 0.013, 0.236 ± 0.074, 0.199 ± 0.006, and 0.196 ± 0.015, respectively (*p* > 0.05). No differences between the groups were noted on day 3 or day 7 (*p* > 0.05). Cellular viability, assessed via CCK-8 assays, showed no significant cytotoxic effects across all CMT concentrations (0.001–1 μg/mL) compared to the control group (0 μg/mL) through day 14 (*p* > 0.05). This finding confirms that CMT preserves cell viability, supporting its application in differentiation protocols (Figure 1C).

### 3.4. Evaluation of SOX9, FAM20B, COL2A1, and COL1A1 Gene Expression with Quantitative Real-Time Polymerase Chain Reaction

**Figure 2:**
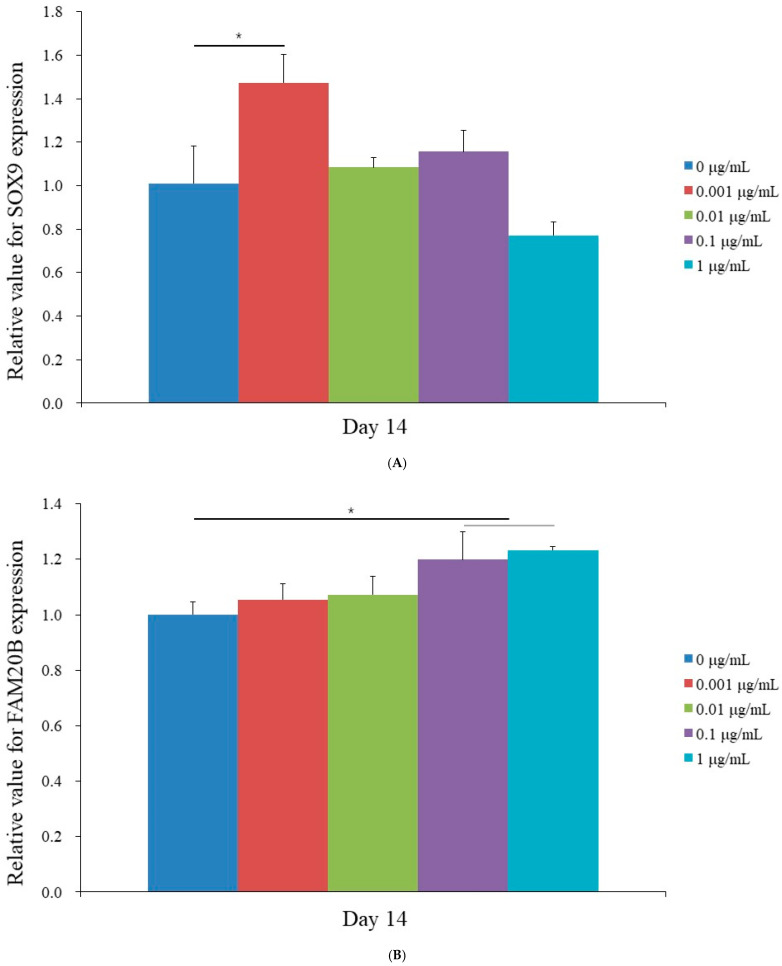

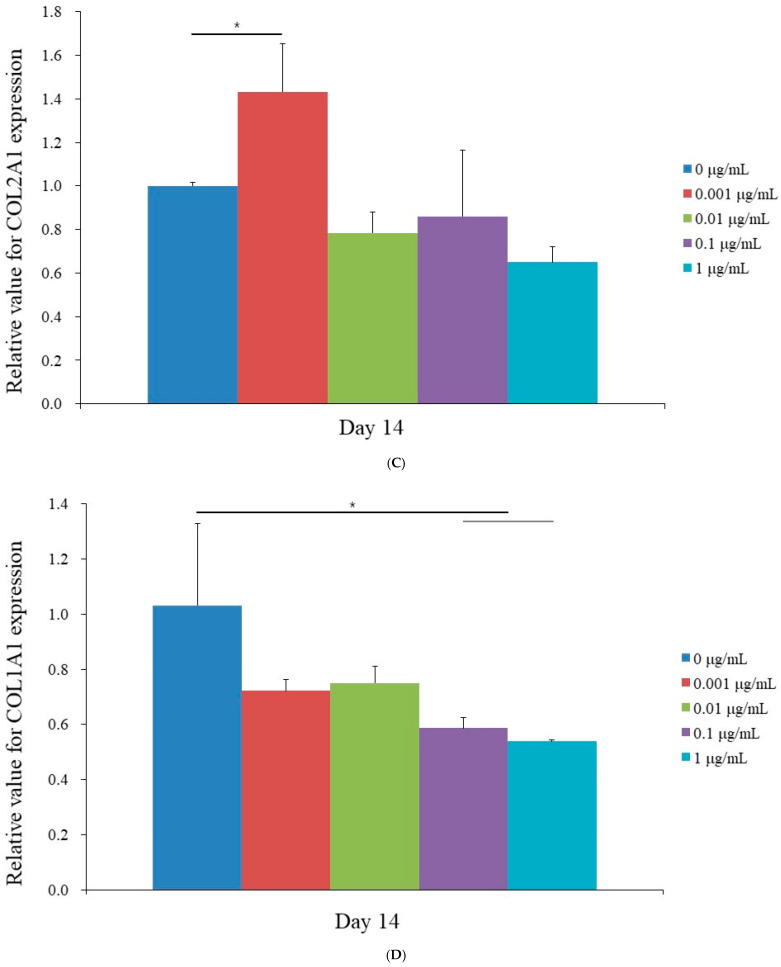
Quantification of gene expression was performed using real-time polymerase chain reaction on day 14 for various concentrations of cumin methanolic extracts. Significant differences compared to the control group are indicated (*). (**A**) The expression of SOX9 was quantified, showing a significant increase in mRNA levels at a concentration of 0.001 μg/mL. (**B**) The expression of FAM20B mRNA was quantified, revealing significant differences in comparison to the control group. (**C**) The expression of COL2A1 mRNA was quantified. (**D**) The expression of COL1A1 was quantified, showing significant differences in mRNA levels compared to the control group.

SOX9 expression showed a significant upregulation at 0.001 μg/mL CMT compared to the control group (0 μg/mL) (*p* < 0.05), indicating enhanced chondrogenic differentiation at this concentration (Figure 2A). Interestingly, the highest concentration (1 μg/mL) led to a decline in SOX9 expression, suggesting a concentration-dependent response. This trend aligns with previous findings highlighting the optimal conditions for differentiation. The mRNA levels of FAM20B were 1.000 ± 0.045, 1.054 ± 0.059, 1.071 ± 0.066, 1.198 ± 0.102, and 1.233 ± 0.012 for CMT concentrations of 0, 0.001, 0.01, 0.1, and 1 μg/mL, respectively (Figure 2B; *p* < 0.05). The mRNA levels of COL2A1 were 1.000 ± 0.015, 1.432 ± 0.222, 0.784 ± 0.096, 0.860 ± 0.304, and 0.649 ± 0.072 for CMT concentrations of 0, 0.001, 0.01, 0.1, and 1 μg/mL, respectively (Figure 2C; *p* > 0.05). In addition, the mRNA levels of COL1A1 were 1.000 ± 0.290, 0.700 ± 0.041, 0.727 ± 0.059, 0.569 ± 0.036, and 0.522 ± 0.007 for CMT concentrations of 0, 0.001, 0.01, 0.1, and 1 μg/mL, respectively (Figure 2D; *p* < 0.05).

### 3.5. Western Blot Analysis

**Figure 3:**
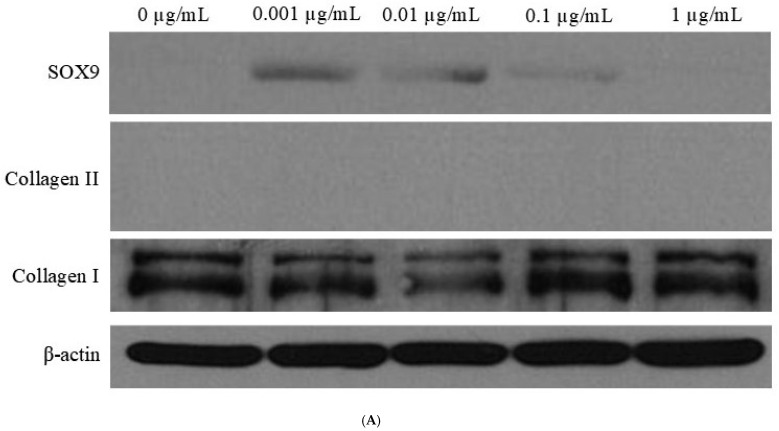

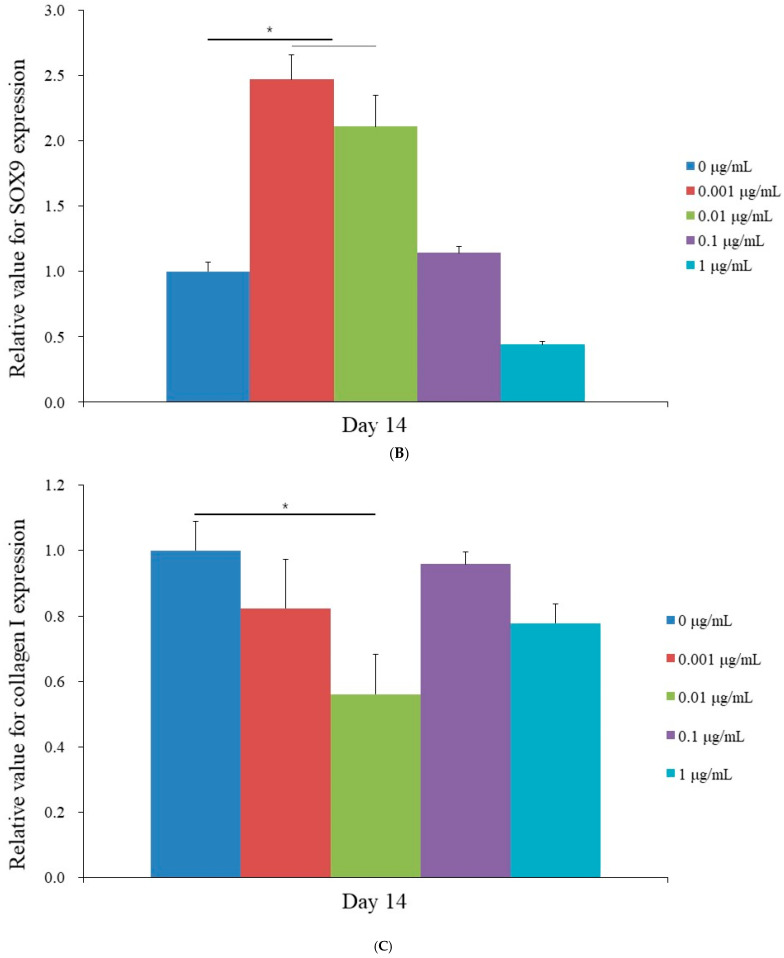
Western blot analysis. (**A**) Western blot analysis to evaluate the expression levels of SOX9, collagen II, collagen I, and β-catenin at different concentrations of cumin methanolic extracts. (**B**) Quantification of expression of SOX9 protein by Western blot analysis on day 14 for different concentrations of cumin methanolic extracts. Significant increases in SOX9 mRNA expression were observed at a concentration of 0.001 and 0.01 μg/mL when compared to the control group (*). (**C**) Quantification of expression of collagen I protein by Western blot analysis on day 14 for different concentrations of cumin methanolic extracts.

Western blot analysis was performed to analyze the protein expression levels of SOX9, collagen II, and collagen I (Figure 3A). Western blot analysis confirmed the concentration-dependent upregulation of SOX9 protein, peaking at 0.001 μg/mL (Figure 3B). This aligns with mRNA expression trends, reinforcing the role of CMT in enhancing chondrogenic differentiation through SOX9 modulation. The level of collagen II expression was low, similar to the undetectable level. Normalization of the protein expression showed collagen I levels of 0.824 ± 0.150, 0.560 ± 0.123, 0.958 ± 0.037, and 0.778 ± 0.059 for CMT at concentrations of 0.001, 0.01, 0.1, and 1 μg/mL, respectively, when the control was considered (1.000 ± 0.090) (Figure 3C). Protein expression analysis confirmed the concentration-dependent effects of CMT on SOX9 levels, with significant upregulation observed at 0.001 and 0.01 μg/mL. These results corroborate mRNA findings and highlight the critical role of post-transcriptional regulation in modulating chondrogenesis (Figure 3B).

### 3.6. Concentration-Dependent mRNA Expression Changes in BMSCs Induced by Cumin: A Clustering and Differential Analysis

We utilized MeV 4.9.0 to perform hierarchical clustering analysis using Euclidean distance correlation and average linkage. Differentially expressed mRNAs were defined by a fold change > 2.0, log2 normalized read counts > 3, and *p*-value < 0.05 to ensure the inclusion of biologically meaningful and statistically significant changes. This analysis identified 76 mRNAs that were differentially expressed across four concentrations of cumin methanolic extract (CMT) compared to the control group (0 µg/mL).

**Figure 4:**
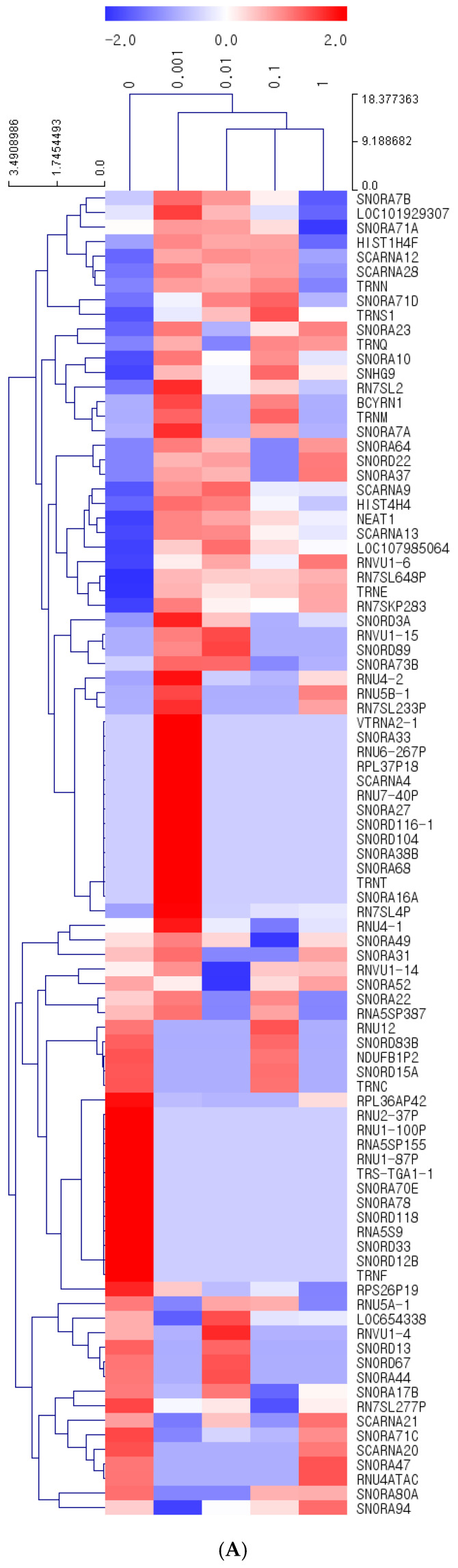

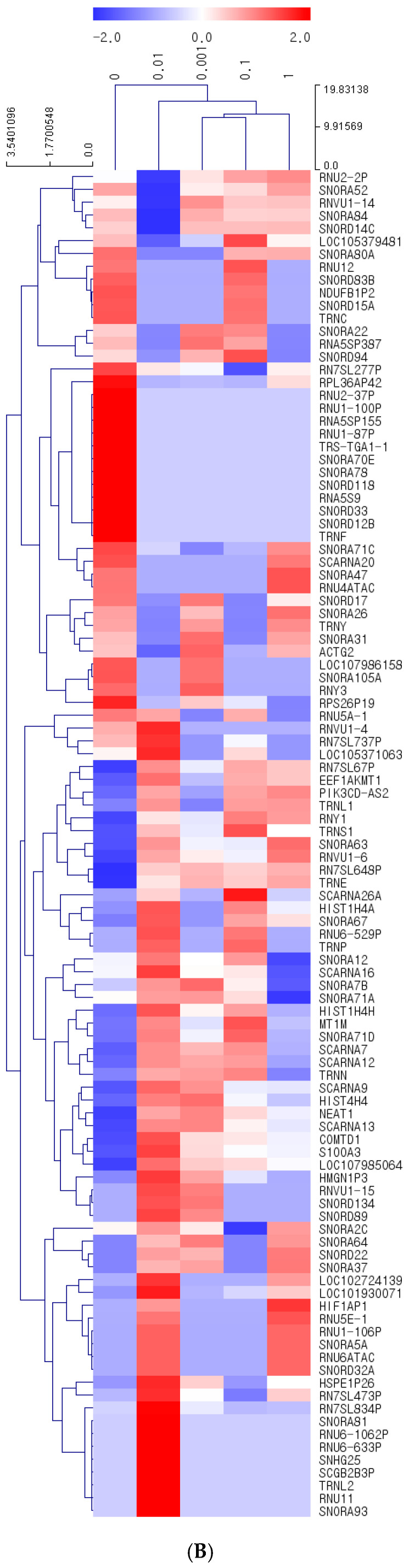

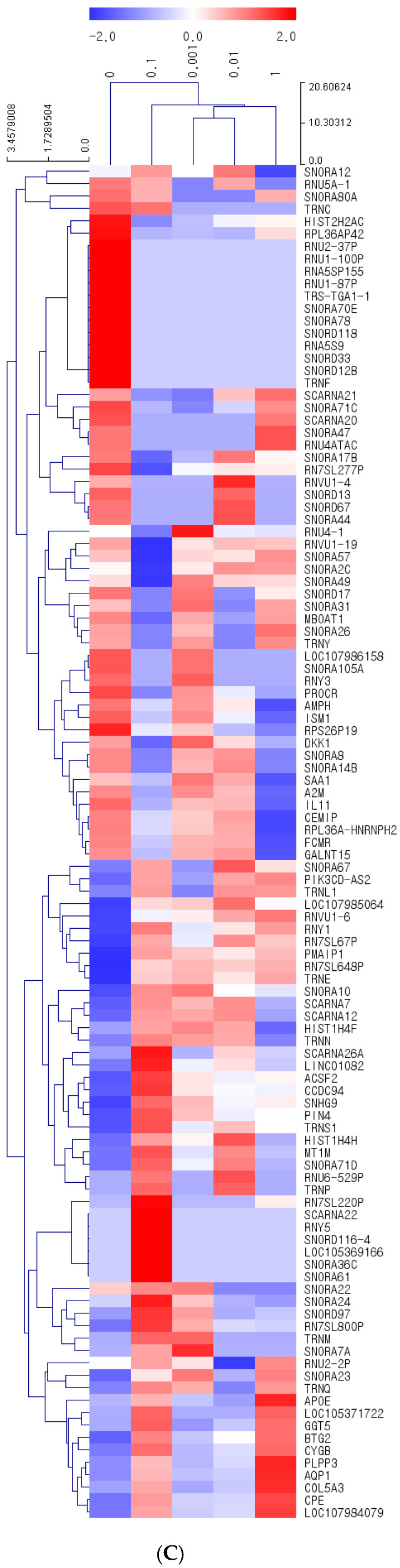

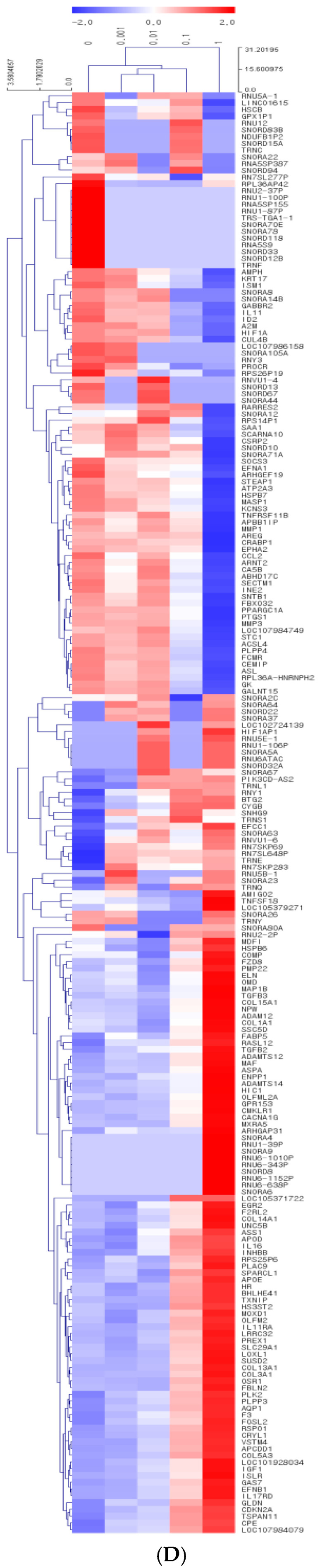
Differentially expressed mRNAs in four different groups. The mRNAs were clustered based on their expression levels, and only those with a fold change greater than 2.0 and a log2 normalized data greater than 3 were included in the analysis. (**A**) The clustering analysis of differentially expressed mRNAs in the 0.001 μg/mL group compared to the control group. (**B**) The analysis of differentially expressed mRNAs in the 0.01 μg/mL group compared to the control group. (**C**) The clustering analysis of differentially expressed mRNAs in the 0.1 μg/mL group vs. the control group. (**D**) The analysis of mRNAs with differential expression in the 1 μg/mL group compared to the control group.

Figure 4A–D illustrates the progressive impact of CMT on mRNA expression, highlighting the concentration-dependent transcriptional response. At the lowest concentration (0.001 µg/mL, Figure 4A), 91 genes exhibited significant changes in expression compared to the control group, increasing to 105 genes at 0.01 µg/mL (Figure 4B), 109 genes at 0.1 µg/mL (Figure 4C), and peaking at 213 genes at 1 µg/mL (Figure 4D). These results underscore the dose-dependent nature of CMT’s effects on gene expression in BMSCs.

**Figure 5:**
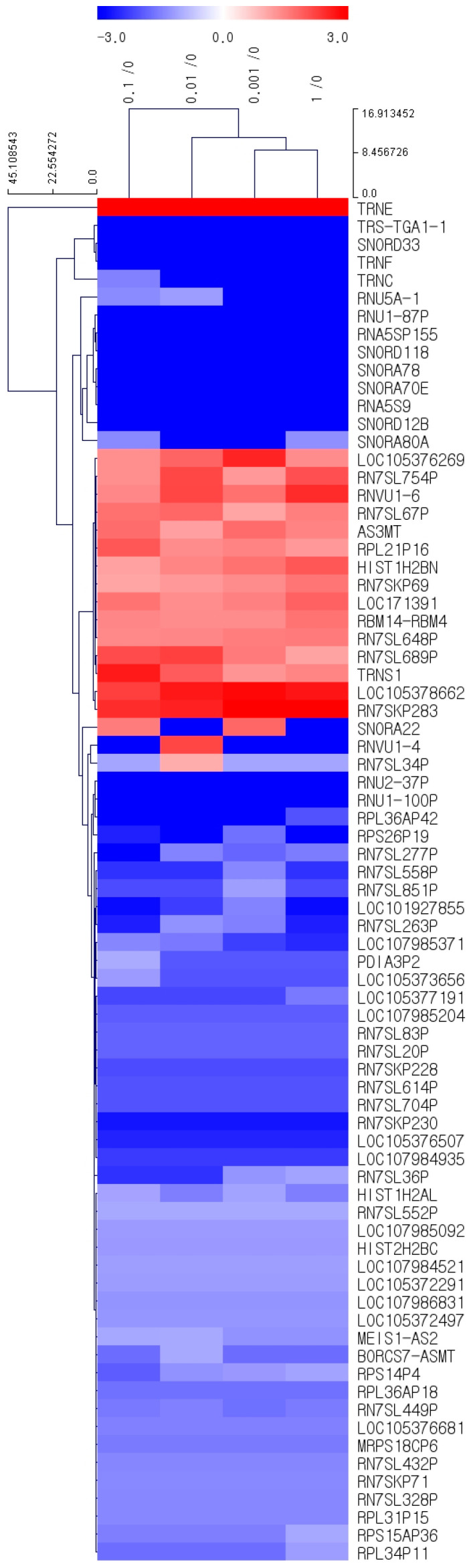
Clustering heatmap of differentially expressed mRNAs in response to cumin methanolic extract (CMT) treatment. The clustering heatmap shows the expression patterns of 76 differentially expressed mRNAs (fold change > 2, *p*-value < 0.05) in bone marrow-derived mesenchymal stem cells (BMSCs) treated with cumin methanolic extract (CMT) at various concentrations (0.001–1 µg/mL) compared to the control group. Upregulated genes are shown in red, downregulated genes in blue, and the intensity of the color represents the magnitude of fold change.

The clustering heatmap provides a visual overview of the 76 genes that met the fold change and statistical thresholds, revealing distinct clusters of upregulated and downregulated genes across CMT concentrations (Figure 5). Upregulated genes (red) were predominantly observed at higher CMT concentrations, with TRNE demonstrating the highest upregulation, with fold changes ranging from +66,034.7 at 0.001 µg/mL to +96,587.8 at 1 µg/mL. Similarly, RN7SKP283 and LOC105378662 showed consistent and significant upregulation across all concentrations. Conversely, downregulated genes (blue), such as SNORA80A, SNORD12B, and TRNC, exhibited robust suppression, indicating potential inhibition of RNA modification pathways.

To provide a more focused perspective, Table 1 highlights the top 50 differentially expressed genes from the 76 identified, ranked by the magnitude of fold change. This table summarizes key regulatory genes, their modulation (upregulated or downregulated), and their functional annotations. Together, the clustering heatmap and Table 1 offer a comprehensive and detailed view of the transcriptional effects of CMT, illustrating its dynamic and concentration-dependent impact on the gene expression landscape in BMSCs.

**Figure 6:**
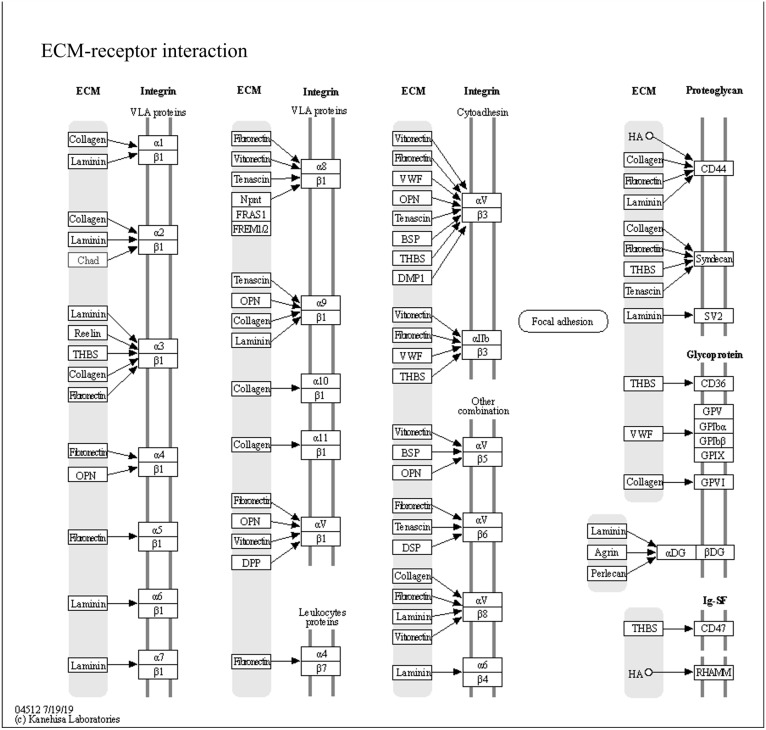
The expression of genes related to chondrogenesis. The extracellular matrix (ECM)receptor interaction pathway map generated using the Kyoto Encyclopedia of Genes and Genomes database. Differentially expressed genes involved in this pathway are highlighted, demonstrating the impact of cumin methanolic extracts on ECM interactions during chondrogenesis.

The results regarding the expression of genes related to chondrogenesis, comparing normalized bone marrow-derived mesenchymal stem cells treated with cumin methanolic extracts to the unloaded group showed that COL1A1, FAM20B, and CHAD displayed an increasing trend. The genes were chosen and added to KEGG for pathway analysis [33,35]. The notable pathway of extracellular matrix (ECM) interaction signaling, which involved the chosen genes, is depicted in Figure 6. These findings suggest that CMT may modulate ECM–receptor interactions, a key mechanism in chondrogenesis. While these trends provide valuable preliminary insights, it is important to note that the results are based on a single experimental replicate, warranting further validation through additional experiments to confirm the reproducibility and statistical significance of these findings. Nonetheless, this analysis lays the groundwork for understanding the molecular mechanisms by which CMT influences chondrogenesis.

## 4. Discussion

This present study aimed to examine the influence of CMT on the chondrogenic differentiation of human mesenchymal stem cells. The results demonstrated that CMT loading promoted enhanced chondrogenic differentiation through the regulation of SOX9 and FAM20B expression. This finding was validated using quantitative real-time polymerase chain reaction and Western blot analysis. SOX9 is a vital regulatory protein that plays a critical role in the various stages of cartilage formation. Its gene expression commences during the skeletal precursor stage and persists throughout the maturation of chondrocytes. The experimental data highlighted that the most crucial determinant of chondrogenic differentiation is the alteration in SOX9 expression, making it a suitable representative gene for this process.

In this study, a diverse range of concentrations, including 0.001, 0.01, 0.1, and 1 μg/mL, were employed to determine the optimal level. The results revealed that there were no discernible effects on the shape and diameter of spheroids between the control and treated groups. The spheroids consistently exhibited a circular morphology. Reducing cell spreading and/or transitioning to a circular phenotype appears to be related to chondrogenesis. In this study, initial assessments, including spheroid morphology, diameter, and cellular viability, were conducted on days 1 and 3 to evaluate the early response of MSC spheroids to CMT. The rationale for using early time points was to confirm the structural stability and cell viability of the spheroids immediately after treatment initiation, ensuring that the spheroids maintained integrity under CMT exposure. These assessments established a baseline of spheroid formation and health, which was crucial for later differentiation-focused analyses. Molecular and protein expression analyses, such as quantitative PCR and Western blotting, were performed on day 14, a time point chosen to align with standard chondrogenic differentiation protocols. MSCs typically require extended culture periods to express differentiation markers like SOX9 and FAM20B at detectable levels. Day 14 provides a more accurate reflection of the chondrogenic effects induced by CMT, as differentiation markers stabilize and increase only after prolonged exposure. This staggered approach of using both early and later time points allowed us to capture both the immediate morphological and viability effects of CMT as well as the more comprehensive differentiation outcomes, aligning with established research practices in 3D MSC spheroid studies [36].

Previous research has shown that the size of the spheroid tends to remain constant even after extracts are added [37,38]. According to a prior report, the ultra-filtrated cumin seed aqueous extract displayed no cytotoxicity at concentrations ranging from 500 to 5000 μg/mL [39]. The minimum inhibitory concentration against Candida albicans was found to be 2–4 µL/mL for cumin, after which the values were compared with the control [40]. The effect of chondrogenesis may vary depending on the experimental model, cell type, and incubation time [41]. Different subpopulations of mesenchymal stem cells exhibit varying chondrogenic potential based on their expression of CD73, CD106, and CD271 markers [42].

Regarding the stability in spheroid size observed at the tested concentrations of CMT, this can be attributed to the differentiation-specific focus of this study. In a 3D culture environment, MSC spheroids naturally prioritize differentiation over proliferation, resulting in stable sizes even when exposed to high concentrations of bioactive compounds. The cell–cell and cell–matrix interactions within spheroids foster structural integrity, which helps maintain a consistent morphology. Additionally, larger spheroids may experience hypoxia and nutrient limitations at their cores, which often restricts growth while allowing for differentiation processes to progress unaffected. The observed stability in spheroid size is therefore consistent with the chondrogenic differentiation focus of this study, where the compound’s primary effects were functional rather than proliferative [43].

The viability of cells can be evaluated using various tetrazolium salts, including MTT, MTS, XTT, and WST [3,44]. In this study, there was no noticeable difference in cellularity between the control and CMT treatment groups. A limitation of this study is that it was conducted in an in vitro setting, and the test concentrations were limited to 0.001 to 1 μg/mL. The unpredictable and unexpected factors that occurred during the experiment caused random deviations and errors, leading to variations and differences in the results of each experiment. However, the positive effects of the tested cumin methanolic extracts persisted throughout the experiment [45]. In this study, the 0 µg/mL CMT group, which contained the same condition as the treated groups but without the extract, served as the control. This ensured that any baseline effects were accounted for. The results showed no significant effects on spheroid morphology, viability, or chondrogenic markers in this group, indicating that methanol at the baseline condition had negligible influence.

Researchers have investigated the use of plant components in chondrogenesis and cartilage repair [17,18]. Pomegranate fruit extract has demonstrated chondrogenic activity, inducing chondrogenic differentiation with increased chondrogenic centers [46]. Aqueous crude extract from Eucomis autumnalis has also been found to induce chondrogenesis [18]. In this study, cumin extract was found to increase the expression of genes associated with chondrogenic differentiation. A variety of extraction methods, including aqueous, methanolic, ethanolic, and ethyl acetate, have been used to extract cumin. Different extraction methods can produce different active components, and the types and amounts of components can vary among different extraction methods [47]. For example, γ-terpinene and ο-cymene have been identified in the essential oil of cumin but not in the hydrosol form [47]. Additionally, terpenoids, which are a major volatile component of cumin, have been reported to be associated with chondrogenic differentiation [48,49]. The methanol extract of cumin contains several monoterpenoid glucosides from the water-soluble portion, and it has been shown that apigenin, quercetin, and luteolin are the major components of flavonoids in cumin [50]. Hypoxia-inducible factor has been reported to be an important mediator for chondrocytes, and some flavonoids, including quercetin, have been associated with hypoxia inducible factor prolyl hydroxylation [51]. The anti-anaphylactic effects of cumin have been evaluated using an aqueous extract [39], and water-soluble polysaccharides from cumin have been found to induce immunostimulatory properties [52]. The methanolic extract of bitter cumin has been tested for antidepressant and anxiolytic activities [53], while the ethanolic extract of cumin has been shown to improve glucose tolerance [54]. The ethyl acetate extract of cumin has been applied to wound healing in animal models [55]. In this study, the methanolic extract of cumin was evaluated for its chondrogenic differentiation potential, and the extraction method was found to impact efficacy and function. Methanol was selected for its high efficiency in extracting both polar and non-polar bioactive compounds, such as flavonoids, terpenoids, and phenolic compounds, which have been shown in previous studies to possess significant protective and neuropharmacological properties, making it the ideal solvent for this experiment [13].

Gene expression analysis has been used to evaluate cartilage regeneration by examining levels of SOX9, COL2, and ACAN [56]. It is reported that SOX9 and Smad signaling pathways play a significant role in chondrogenic and chondroprotective effects [17]. Mesenchymal stem cells’ chondrogenic differentiation is regulated by the extracellular signal-regulated kinase and c-Jun N-terminal kinase pathways [57,58]. In that study, SOX9 and COL2 expression was evaluated through quantitative real-time polymerase chain reaction and Western blot analysis. SOX9, as a critical factor in chondrogenesis, promotes the differentiation of mesenchymal stem cells into chondrocytes, and it may work in conjunction with other transcription factors, such as SOX6, to activate the expression of cartilage matrix proteins, including collagen type II [59]. FAM20B, a protein involved in the biosynthesis and modification of glycosaminoglycans, which are components of the extracellular matrix in cartilage, is crucial in maintaining the homeostasis of the cartilage matrix in adult cartilage [60]. CHAD, a small leucine-rich proteoglycan, is part of the extracellular matrix of cartilage and reportedly facilitates the adhesion of chondrocytes to the extracellular matrix, as well as plays a role in maintaining the structure and integrity of cartilage tissue [61,62]. The results of this study demonstrated that CMT treatment can enhance chondrogenic differentiation by regulating the expression of SOX9, FAM20B, and CHAD genes. In addition to the primary findings related to chondrogenic differentiation, this study observed a differential expression of RNU 1-4, which may reflect an adaptive cellular response to *Cuminum cyminum* treatment. RNU 1-4, as small nuclear RNAs (snRNAs), are essential for the splicing of pre-mRNAs and the maturation of other non-coding RNAs, making them critical to gene regulation under specific cellular conditions. Alterations in snRNA levels can influence the efficiency of pre-mRNA splicing, potentially modulating the expression of downstream genes involved in stem cell differentiation and metabolism. This mechanism aligns with findings from previous research, where snRNAs were shown to participate in regulatory responses to treatment and environmental stress, impacting alternative splicing and gene expression pathways related to differentiation and cellular adaptation [63].

Furthermore, this study identified small nucleolar RNAs (snoRNAs) as differentially expressed in response to *Cuminum cyminum* treatment. SnoRNAs, typically involved in the maturation and modification of ribosomal RNA (rRNA), are increasingly recognized as regulators in gene expression and cellular stress responses. The observed differential expression of snoRNAs may indicate an adaptive cellular mechanism wherein snoRNAs respond to external compounds, such as bioactive plant extracts, to regulate RNA stability, translation, and ribosomal biogenesis. Emerging research suggests that snoRNAs can serve as biomarkers, reflecting cellular responses to metabolic changes and therapeutic agents, and thereby influencing downstream pathways involved in cellular differentiation and metabolism [64].

The possible explanations for the discrepancies in the findings may be attributed to differences in the mRNA and protein levels over time. It is important to note that some of the outcomes contradicted those obtained from real-time PCR and Western blot analysis, which underscores the need for additional research, including in vivo study.

## 5. Conclusions

In summary, the findings of this investigation revealed that chondrogenic differentiation can be enhanced through the treatment of CMT by regulating the expression of SOX9 and FAM20B genes, as demonstrated by both mRNA sequencing and protein expression analysis. It is suggested that SOX9 plays a crucial role in CMT-induced chondrogenic differentiation. The use of CMT may prove to be a promising approach for promoting cartilage repair and may have significant clinical implications for the treatment of joint diseases. However, further research is necessary to fully understand the precise mechanisms by which CMT affects chondrogenic differentiation and to explore its potential clinical applications in greater detail.

## Figures and Tables

**Table 1:** The top 50 genes from the 76 identified as differentially expressed across different concentrations of cumin methanolic extract (CMT) (fold change > 2 and *p*-value < 0.05).

Gene Symbol *	Gene Name	Modulation	Description
*TRNE*	Transfer RNA Glutamic Acid	Upmodulated	Transfer RNA gene
*RN7SKP283*	7SK Small Nuclear RNA Pseudogene 283	Upmodulated	RNA-binding protein
*LOC105378662*	Uncharacterized LOC105378662	Upmodulated	Non-coding RNA
*LOC105376269*	Uncharacterized LOC105376269	Upmodulated	Non-coding RNA
*SNORA22*	Small Nucleolar RNA, H/ACA Box 22	Downmodulated	Small nucleolar RNA
*AS3MT*	Arsenite Methyltransferase	Upmodulated	Arsenite methyltransferase
*RNVU1-6*	RNA Vault 1-6	Upmodulated	RNA, vault structure
*HIST1H2BN*	Histone Cluster 1 H2B N	Upmodulated	Histone-related protein
*RN7SL689P*	Signal Recognition Particle RNA Pseudogene 689	Upmodulated	Ribosomal pseudogene
*RN7SL648P*	Signal Recognition Particle RNA Pseudogene 648	Upmodulated	Ribosomal pseudogene
*LOC171391*	Uncharacterized LOC171391	Downmodulated	Non-coding RNA
*RPL21P16*	Ribosomal Protein L21 Pseudogene 16	Upmodulated	Ribosomal pseudogene
*RN7SKP69*	7SK Small Nuclear RNA Pseudogene 69	Downmodulated	Small nuclear RNA
*RBM14-RBM4*	RNA Binding Motif Protein 14	Upmodulated	RNA-binding protein
*TRNS1*	Transfer RNA Synthetase 1	Upmodulated	Transfer RNA synthetase
*RN7SL754P*	Signal Recognition Particle RNA Pseudogene 754	Downmodulated	Ribosomal pseudogene
*RN7SL67P*	Signal Recognition Particle RNA Pseudogene 67	Upmodulated	Ribosomal pseudogene
*RN7SL552P*	Signal Recognition Particle RNA Pseudogene 552	Downmodulated	Ribosomal pseudogene
*RN7SL34P*	Signal Recognition Particle RNA Pseudogene 34	Upmodulated	Ribosomal pseudogene
*HIST1H2AL*	Histone Cluster 1 H2A L	Downmodulated	Histone-related protein
*RN7SL851P*	Signal Recognition Particle RNA Pseudogene 851	Downmodulated	Ribosomal pseudogene
*LOC107984521*	Uncharacterized LOC107984521	Upmodulated	Non-coding RNA
*LOC105372291*	Uncharacterized LOC105372291	Downmodulated	Non-coding RNA
*LOC107985092*	Uncharacterized LOC107985092	Downmodulated	Non-coding RNA
*HIST2H2BC*	Histone Cluster 2 H2B C	Upmodulated	Histone-related protein
*RPS14P4*	Ribosomal Protein S14 Pseudogene 4	Downmodulated	Ribosomal pseudogene
*LOC105372497*	Uncharacterized LOC105372497	Upmodulated	Non-coding RNA
*RN7SL36P*	Signal Recognition Particle RNA Pseudogene 36	Downmodulated	Ribosomal pseudogene
*LOC107986831*	Uncharacterized LOC107986831	Downmodulated	Non-coding RNA
*MEIS1-AS2*	MEIS1 Antisense RNA 2	Downmodulated	Non-coding RNA
*RN7SKP71*	7SK Small Nuclear RNA Pseudogene 71	Upmodulated	Small nuclear RNA
*RN7SL328P*	Signal Recognition Particle RNA Pseudogene 328	Downmodulated	Ribosomal pseudogene
*RPL31P15*	Ribosomal Protein L31 Pseudogene 15	Upmodulated	Ribosomal pseudogene
*RN7SL558P*	Signal Recognition Particle RNA Pseudogene 558	Downmodulated	Ribosomal pseudogene
*RN7SL432P*	Signal Recognition Particle RNA Pseudogene 432	Downmodulated	Ribosomal pseudogene
*LOC101927855*	Uncharacterized LOC101927855	Downmodulated	Non-coding RNA
*RN7SL263P*	Signal Recognition Particle RNA Pseudogene 263	Upmodulated	Ribosomal pseudogene
*LOC105376681*	Uncharacterized LOC105376681	Upmodulated	Non-coding RNA
*RPS15AP36*	Ribosomal Protein S15A Pseudogene 36	Downmodulated	Ribosomal pseudogene
*MRPS18CP6*	Mitochondrial Ribosomal Protein S18C Pseudogene 6	Upmodulated	Mitochondrial pseudogene
*RPL36AP18*	Ribosomal Protein L36A Pseudogene 18	Upmodulated	Ribosomal pseudogene
*RPS26P19*	Ribosomal Protein S26 Pseudogene 19	Downmodulated	Ribosomal pseudogene
*RN7SL449P*	Signal Recognition Particle RNA Pseudogene 449	Downmodulated	Ribosomal pseudogene
*RPL34P11*	Ribosomal Protein L34 Pseudogene 11	Upmodulated	Ribosomal pseudogene
*BORCS7-ASMT*	BORCS7 Antisense RNA, Methyltransferase	Downmodulated	Non-coding RNA
*RN7SL277P*	Signal Recognition Particle RNA Pseudogene 277	Upmodulated	Ribosomal pseudogene
*RN7SL20P*	Signal Recognition Particle RNA Pseudogene 20	Upmodulated	Ribosomal pseudogene
*RN7SL83P*	Signal Recognition Particle RNA Pseudogene 83	Upmodulated	Ribosomal pseudogene
*LOC107985204*	Uncharacterized LOC107985204	Downmodulated	Non-coding RNA
*PDIA3P2*	Protein Disulfide Isomerase A3 Pseudogene 2	Upmodulated	Ribosomal pseudogene

* These genes were ranked by the magnitude of their fold changes and are presented along with their modulation status (upregulated or downregulated) and functional descriptions where available. This subset provides a focused perspective on the most significant transcriptional changes induced by CMT treatment.

## Data Availability

All data analyzed during this study are included in this published article, further inquiries can be directed to the corresponding authors.
